# New Advertisements

**Published:** 1886-04-20

**Authors:** 


### NEW ADVERTISEMENTS.

ADVANCE COURIER, etc., Chicago, Ill. Read their advertisement in this journal.

JACOBS' PHARMACY.—Don't fail to read the advertisement of Jacobs' Pharmacy, commencing with this number of our journal. They are live men, clever, enterprising and accommodating. Their place of business is very central and convenient, on corner of Marietta and Peachtree.

SEE new advertisements from the following excellent houses. We regret the want of space to refer to them more fully: Charles Truax & Co., medical supplies, instruments, etc. John Barry, clinical thermometers. Heyden's Viburnum Compound. Wm. E. Richards, stock, grain and oil.

☞ Receipts will appear in our next.

